# Comprehensive molecular analysis of 26 newly established human pancreatic ductal adenocarcinoma cell lines reveals two clusters with variating drug sensitivities

**DOI:** 10.1186/s12935-025-03671-8

**Published:** 2025-02-19

**Authors:** Ju Eun Maeng, Jae-Hyeon Kim, Soon-Chan Kim, Won-Gun Yun, Wooil Kwon, Youngmin Han, Do-Youn Oh, Sang Hyub Lee, Jin-Young Jang, Ja-Lok Ku

**Affiliations:** 1 Department of Biomedical Sciences, Seoul National University College of Medicine, Seoul, 03080 Korea; 2 Laboratory of Cell Biology, Cancer Research Institute, Seoul National University College of Medicine, 101, Daehak-Ro, Jongno-Gu, Seoul, 03080 Korea; 3 Department of Surgery, Seoul National University College of Medicine, 103 Daehak-Ro, Jongno-Gu, Seoul, 03080 Korea; 4 Department of Internal Medicine, Seoul National University Hospital, Seoul, 03080 Korea; 5 Ischemic/Hypoxic Disease Institute, Seoul National University College of Medicine, Seoul, 03080 Korea

## Abstract

**Background:**

Pancreatic ductal adenocarcinoma (PDAC) is a malignant form of cancer with the worst survival rate and an extremely low rate of response to treatments. The development and molecular characterization of pancreatic cancer cell lines (PCCLs) are essential for studying the biology of highly aggressive pancreatic adenocarcinoma.

**Methods:**

We applied whole exome sequencing (WES) and RNA-seq to identify molecular characteristics of 26 newly established PCCLs. Eighteen clinically relevant anti-cancer drugs were used to assess highly heterogeneous drug responses across the 26 cell lines.

**Results:**

We confirmed that common driver mutations of PDAC were well retained in our cell lines through WES analysis. Transcriptomic analysis identified two representative clusters that correlated with responses to certain drugs. By using Moffitt’s classification method, the two clusters, C1 and C2, showed comparable expression patterns to “Basal-like” and “Classical” types, respectively. Drug screening results showed varying responses among different cell lines. In our cohort, C2 displayed greater sensitivity to anti-cancer drugs compared to C1. Furthermore, drugs targeting similar molecular pathways exhibited corresponding reactions among cell lines.

**Conclusions:**

Our results underscored that transcriptomic features of pancreatic cancer correlate with drug sensitivity rather than with the effects of targeted drugs. Cell lines are useful in vitro model systems for studying the molecular mechanisms of PDAC.

**Graphical Abstract:**

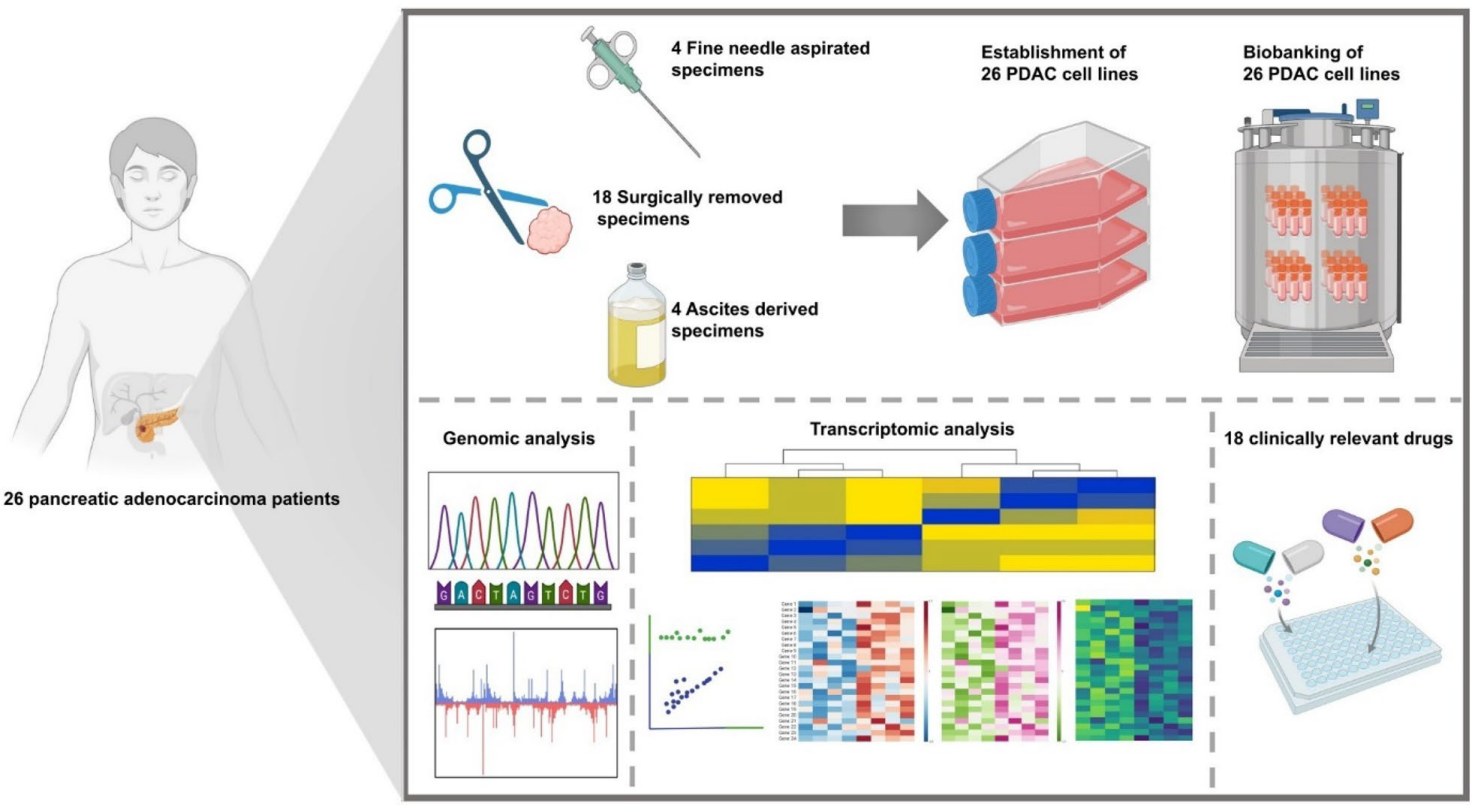

**Supplementary Information:**

The online version contains supplementary material available at 10.1186/s12935-025-03671-8.

## Introduction

Pancreatic cancer is a highly lethal disease, ranking as the seventh leading cause of cancer-related death globally [1]. Patients with pancreatic ductal adenocarcinoma (PDAC) have an extremely low 5-year survival rate of approximately 9% [2]. PDAC is associated with a number of factors that contribute to its poor prognosis, including advanced stage diagnosis and resistance to chemotherapy [3]. To enhance the prognosis of PDAC, the establishment of early identification methods and successful treatments is necessary [4]. Chemotherapy remains the mainstay of treatment for patients with PDAC due to *KRAS* mutations and the loss of tumor suppressor genes [5, 6].

Repeated genomic mutations in *KRAS* have been identified in more than 90% of PDACs [7, 8]. In addition, mutations in tumor suppressor genes, including *TP53, CDKN2A*, and *SMAD4* have been discovered in 72%, 30%, and 32% of cases, respectively [9]. Genetic mutations existing in PI3K pathways and other related genes cause tumor formation in PDAC. At least 3% of pancreatic cancer patients carry *PIK3CA* gene mutations, which result in tumorigenesis and contribute to tumor proliferation [10]. The characterization of PDAC and the study of its genetic variations have led to a better understanding of this disease and form the basis for improving its early detection and advanced treatments. Organizations worldwide offer 69 human pancreatic cancer cell lines for distribution; however, there is a need for a diverse range of cell lines to strengthen the efficacy of in vitro model research.

To address the need for new therapeutic approaches based on in-depth transcriptomic analysis, we established 26 pancreatic cancer cell lines (PCCLs), which were subjected to extensive multi-omics analysis as well as high-throughput screening. Previous studies have shown that PDAC can be divided into distinct clusters through transcriptomic profiling [11–13]. In our study, we integrated multi-layered data centered on the transcriptomic clustering. Our results indicated that the two distinct groups largely separated other layers of omics, including genomic mutations and drug responses.

## Methods

### Establishment and maintenance of human pancreatic cancer cell lines

The research was reviewed and approved by the Institutional Review Board of Seoul National University Hospital (IRB No. 1102-098-357). Written informed consent was obtained from all patients enrolled in this study.

Clinical samples used for cell line establishment were obtained from patients at Seoul National University and pancreatic tumor specimens were collected by fine needle aspiration, surgical resection, or ascites puncture. A total of 26 cell lines from pathologically proven pancreatic adenocarcinomas were established. Solid tumors were finely minced with scissors and dispersed into small aggregates. Appropriate amounts of fine neoplastic tissue fragments were seeded into T-25cm^2^ flasks (Corning, NY, USA, Cat#353108). Most of the tumor cells were initially cultured in Opti-MEM I (Thermo Fisher Scientific, MA, USA) supplemented with 5% fetal bovine serum (GE Healthcare Life Sciences, Buckinghamshire, UK). Confined-area trypsinization or scraping method was used to attain a pure tumor cell population when stromal cells like mesothelial cells or fibroblasts grew in the initial culture. Established cell lines were sustained in RPMI 1640 medium with 10% fetal bovine serum and 1% (v/v) penicillin and streptomycin (10,000U/ml). Cell lines were cultivated in humidified incubators at 37 °C in an atmosphere of 5% CO2 in air. Organoid derived cell lines were retrieved from established patient-derived organoids and passaged with identical cell line medium used for cell lines. The initial passage was assigned when tumor cell growth was observed. Repeatedly expanding cell cultures were further passaged with trypsinization. For culture populations containing both floating and adherent cells, floating cells were spun down by centrifugation at 1500 rpm for 3 min and dispersed by pipetting. Established cell lines in this study will be deposited at Korean Cell Line Bank (http://cellbank.snu.ac.kr) for distribution to researchers worldwide.

### Cell growth properties

At 70–90% confluency, cells were detached from a T75 flask with 1X trypsin. After centrifugation, the cells were re-suspended with culture medium. Cell suspensions containing 5 × 10^4^ to 2 × 10^5^ cells were seeded into 96-well culture plates in 80 μL of complete culture medium and incubated at 37 °C in an atmosphere of 5% CO2 in air. Since the first cell seeding, CellTiter-Glo® (Promega) was added to each well every 24 h. After 10 min of adding CellTiter-Glo®, the optical density was measured by using Luminoskan Ascent™ (Thermo Fisher Scientific, MA, USA) at 450 nm wavelength. Cell numbers were measured at 24-h intervals for at least 5 days. The morphology of each cell lines was observed with phase-contrast microscopy. Mycoplasma contamination was tested using the 16S-rRNA gene-based PCR amplification method with the e-Myco Mycoplasma PCR detection kit (Intron Biotechnology, Gyeonggi, Korea).

### Nucleic acid isolation and complementary DNA synthesis

Genomic DNA was extracted from the cell lines using QIAamp DNA Mini Kit (Qiagen, Hilden, Germany) and RNA was extracted by using TRIzol (Life technologies, CA, USA) and RNeasy Plus Mini Kit (Qiagen, Hilden, Germany) according to the manufacturer’s protocol.

### DNA fingerprinting analysis using 15 short tandem repeat (STR) loci and amelogenin marker

The genomic DNA from each cell line was amplified using an AmpFlSTR identifier polymerase chain reaction (PCR) amplification kit (Applied Biosystems, CA, USA). A single cycle of PCR amplified 15 short tandem repeat markers (CSF1PO, D2S1338, D3S1358, D5S818, D7S820, D8S1179, D13S317, D16S539, D18S51, D19S433, D21S11, FGA, TH01, TPOX and vWA) and an amelogenin gender-determining marker containing highly polymorphic microsatellite markers. Amplified PCR products were analyzed by an ABI 3500XL Genetic analyzer (Applied Biosystems, CA, USA).

### Drug sensitivity test and analysis

Preparation of cell suspensions was previously described. According to various growth rates, 2–8 × 10^5^ cells/mL were seeded onto 96-well tissue culture plates in 80 μL of complete culture medium and incubated in humidified incubators at 37 °C for 24 h in an atmosphere of 5% CO_2_ and 95% air. 18 anti-cancer agents were serially diluted and then added to each well with a volume of 20 μL. After 72 h of incubation, CellTiter-Glo® (Promega, WI, USA) was added to each well according to the manufacturer’s instructions. After 20 min of incubation at 37 °C, the optical density was measured at fluorescence using Luminoskan Ascent™ (Thermo Fisher Scientific, MA, USA). To evaluate drug test results between established cell lines, area under the curve (AUC) was used and analyzed using R program version 4.2.2 (R Foundation for Statistical Computing, Vienna, Austria). The same method was used for MRTX1133 and the results were analyzed with GraphPad Prism 5 (GraphPad Software, CA, USA).

### Whole exome sequencing

Whole exome sequencing was conducted by DNALink (Seoul, Republic of Korea). Whole-exome capture was performed on all samples with the SureSelect Human All Exon V5 Kit (Agilent Technologies, Tokyo, Japan) using the Bravo automated liquid handler. The captured targets were sequenced on a HiSeq 2500 platform (Illumina, San Diego, CA, USA) with paired-end 100 bp reads. Briefly, paired-end sequences were mapped to the human genome reference sequence (UCSC assembly hg19, based on the original GRCh37 from NCBI) using BWA-MEM (version 0.7.12), which generated BAM format mapping result files. PCR duplicates were removed using Picard tools (2.2.1) with the MarkDuplicates function. Base quality score recalibration (BQSR) and local realignment around indels were carried out sing the Genome Analysis Toolkit (GATK). Variant genotyping was performed for each sample using GATK HaplotypeCaller (GATKv3.5.0) based on the previously generated BAM files, enabling the detection of SNPs and short indels. The resulting variants were annotated using SnpEff (version 4.2) and outputted in VCF file format. Variants were filtered against dbSNP and the 1000 Genomes project database. Additional filtering was performed using databases such as ClinVar, and dbSNP (138) with SnpEff to refine the variant list further.

### RNA sequencing and fusion gene analysis

Total RNA was isolated from cell lysate using TRIzol (Qiagen, Hilden, Germany) and Qiagen RNeasy kit (Qiagen, Hilden, Germany). Sequencing libraries were prepared using the Illumina TruSeq stranded total RNA library prep kit. Fifty-one million reads were obtained from the cell lysates. Following base-calling and alignment with the Tuxedo Suite, the rejected reads were analyzed using FusionMap, ChimeraScan, and Defuse with default parameters for RNA and alignment to GRCh37.72. The fusion gene extracted from FusionMap and Defuse was sorted through fusionGDB, fusion gene annotation data base. SNU-4405, an outlier, was removed from analysis for a better distribution.

### Protein isolation and western blotting

Cells were harvested with a cell scraper after washing with cold PBS. Whole protein was extracted with EzRIPA buffer (ATTO Co., Tokyo, JAPAN) supplied with 1% protease inhibitor and 1% of phosphatase inhibitor. The protein concentration was calculated by Pierce™ BCA Protein Assay Kit (Thermo Fisher Scientific, MA, USA), then equal amounts of protein were loaded on 4%−15% Mini-PROTEAN TGX Precast Gels (BIO-RAD, Hercules, CA) and blotted at 50 V for 2 h. Proteins were transferred to a Trans-Blot Turbo Transfer Pack (BIO-RAD) using the Trans-Blot Turbo Transfer System V1.02 machine (BIO-RAD) at 2.5 A and 25 V. The membrane was incubated in 1.5% skim milk containing 0.5% Tween 20 for an hour at room temperature. Primary antibodies against KRAS (Abcam, Cambridge, UK) (1:500), EGFR (Cell Signaling Technology, Danvers, MA, USA) (1:1000), Phospho-EGFR-Tyr1068 (Cell Signaling Technology, Danvers, MA, USA) (1:1000), HER2 (Cell Signaling Technology, Danvers, MA, USA) (1:1000), ERK (Applied Biological Materials Inc., Richmond, BC, Canada) (1:1000), Phospho-ERK-Thr202/Tyr204 (Cell Signaling Technology, Danvers, MA, USA) (1:1000), PTEN (Cell Signaling Technology, Danvers, MA, USA) (1:1000), mTOR (Cell Signaling Technology, Danvers, MA, USA) (1:1000), Phospho-mTOR-Ser2448 (Cell Signaling Technology, Danvers, MA, USA) (1:1000), Akt (Cell Signaling Technology, Danvers, MA, USA) (1:1000), Phospho-Akt-Thr308 (Cell Signaling Technology, Danvers, MA, USA) (1:500), β-actin (Invitrogen, Carlsbad, CA, USA) (1:1000) were introduced to the membrane and incubated at room temperature for 1 h. Peroxidase-conjugated mouse or rabbit IgG antibody (Jackson Immunoresearch, West Grove, PA, USA) (1:5000) was added as a secondary antibody and incubated at room temperature for 1 h. After chemiluminescent working solution, SuperSignal™ West Pico PLUS (Thermo Fisher Scientific, MA, USA), was decanted to the membrane. The membrane was exposed to Fuji RX film for 1–5 min.

### Mutation profiling and enriched pathway analysis

All analysis was performed using R program version 3.6.3 (R Foundation for Statistical Computing, Vienna, Austria). Mutation profiling of whole exome sequencing was performed using ComplexHeatmap (v2.2.0) package [14]. To identify known pathogenic/likely pathogenic mutations, we searched for what was reported in Clinvar database (https://www.ncbi.nlm.nih.gov/cinvar). ConsensusClusterPlus package was used to find the optimal number of clusters from total raw read counts of transcripts [15]. For further analysis, DESeq2 package was used to compare between samples with C1 and C2 in order to compute log twofold changes and corresponding p-values for each gene [16]. PCA loadings was generated by using PCA tools package. Pathway enrichment analysis was performed using tools such as clusterProfiler, pathfindR, GSVA, and WGCNA.

## Results

### General characteristics of cell lines

**Fig. 1:**
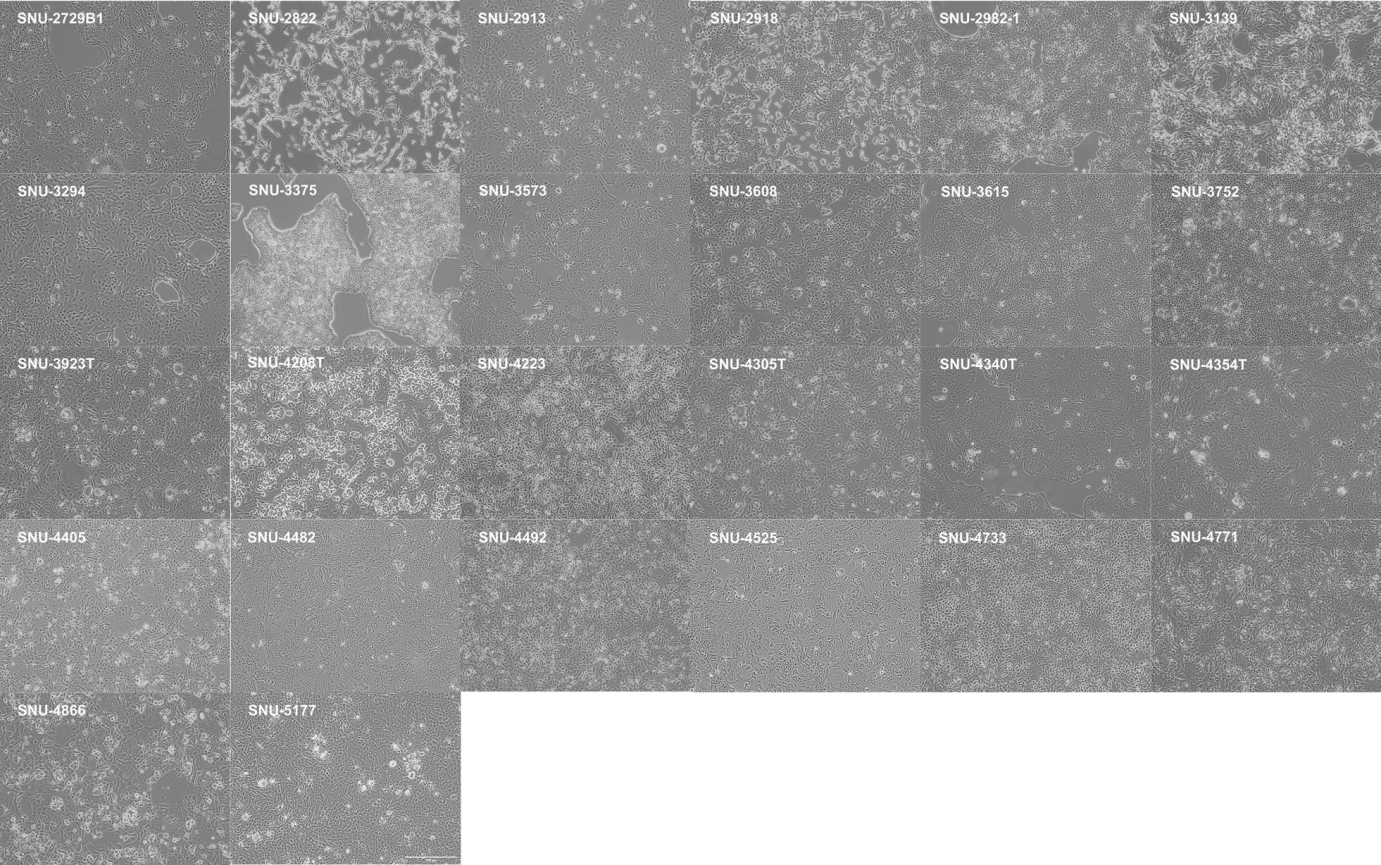
Brightfield images of 26 human pancreatic cancer cell lines showed polygonal, fibroblast-like, oval, and round morphological attributes. Scale bar: 100 μm

The clinical characteristics of the established cell lines are summarized in Table 1. The cell lines displayed distinct morphological characteristics, such as polygonal, fibroblast-like, and oval shapes (Fig. 1). The doubling times of the cell lines ranged from 22.6 to 119.5 h (Table 1). DNA fingerprinting analysis identified a heterogeneous distribution of 15 tetranucleotide repeat loci and the Amelogenin gender determining marker in each cell line. It was confirmed that all 26 cell lines were free from cross-contamination (Additional file 1: Table. S1). All cell lines were also confirmed to be free of mycoplasma contamination (Additional file 1: Fig. S1). Cell lines introduced in this study will be deposited in Korean Cell Line Bank (http://cellbank.snu.ac.kr) for distribution to researchers worldwide.

**Table 1:** Clinicopathological table

No.	Cell line	Sex	Age	Derived origin	Growth pattern	Doubling time	Cell morphology	Clinical stage	First treatment	Chemotherapy regimen (RESPONSE)	Surgery
1	SNU-2729B1	M	46	Tissue	Adherent	50.4	Polygonal	Metastatic	Chemotherapy	Gemcitabine/Erlotinib (Progressive disease)	Not applicable
2	SNU-2822	F	68	Ascites	Adherent	60.7	Fibroblast-like	Metastatic	Chemotherapy	Gemcitabine/Cisplatin (Stable disease)	Not applicable
3	SNU-2913	M	55	Ascites	Adherent	22.6	Fibroblast-like/Polygonal	Metastatic	Chemotherapy	FOLFIRINOX (Progressive disease)	Not applicable
4	SNU-2918	F	61	Ascites	Adherent	75	Oval	Resectable	Surgery	Not applicable	Pancreaticoduodenectomy
5	SNU-2982-1	F	71	Ascites	Adherent	66	Polygonal	Locally advanced	Chemotherapy	FOLFIRINOX (Stable disease)	Not applicable
6	SNU-3139	M	78	Tissue	Adherent	77.2	Polygonal	Locally advanced	Chemotherapy	5-fluorouracil (Stable disease)	Not applicable
7	SNU-3294	M	66	Tissue	Adherent	85.4	Polygonal	Borderline resectable	Surgery	Not applicable	Pancreaticoduodenectomy
8	SNU-3375	M	55	Tissue	Adherent	52.3	Round	Resectable	Surgery	Not applicable	Distal pancreatectomy
9	SNU-3573	M	49	Tissue	Adherent	67.6	Polygonal	Metastatic	Chemotherapy	FOLFIRINOX (Partial response)	Not applicable
10	SNU-3608	M	76	Tissue	Adherent	59.5	Polygonal	Resectable	Surgery	Not applicable	Distal pancreatectomy
11	SNU-3615	M	77	Tissue	Adherent	68.4	Oval/Round	Resectable	Surgery	Not applicable	Pancreaticoduodenectomy
12	SNU-3752	F	70	Tissue	Adherent	113	Polygonal/Round	Resectable	Surgery	Not applicable	Distal pancreatectomy
13	SNU-3923T*	M	69	Tissue	Adherent	80.4	Round	Resectable	Surgery	Not applicable	Pancreaticoduodenectomy
14	SNU-4208T*	M	45	Biopsy	Adherent	61.9	Round	Metastatic	Chemotherapy	FOLFIRINOX (Progressive disease)	Not applicable
15	SNU-4223	F	47	Tissue	Adherent	79.3	Polygonal	Borderline resectable	Surgery	Not applicable	Pancreaticoduodenectomy
16	SNU-4305T*	F	59	Biopsy	Adherent	45.1	Polygonal	Locally advanced	Chemotherapy	FOLFIRINOX (Stable disease)	Not applicable
17	SNU-4340T*	M	66	Biopsy	Adherent	85.1	Polygonal	Metastatic	Chemotherapy	FOLFIRINOX (Stable disease)	Not applicable
18	SNU-4354T*	F	64	Biopsy	Adherent	73.2	Polygonal	Locally advanced	Chemotherapy	FOLFIRINOX (Progressive disease)	Not applicable
19	SNU-4405	F	61	Tissue	Adherent	119.5	Polygonal	Resectable	Surgery	Not applicable	Distal pancreatectomy
20	SNU-4482	M	65	Tissue	Adherent	48.6	Polygonal	Resectable	Surgery	Not applicable	Total pancreatectomy
21	SNU-4492	F	80	Tissue	Adherent	37.9	Oval/Round	Resectable	Surgery	Not applicable	Distal pancreatectomy
22	SNU-4525	M	56	Tissue	Adherent	78.2	Oval	Resectable	Surgery	Not applicable	Distal pancreatectomy
23	SNU-4733	M	61	Tissue	Adherent	63.8	Polygonal	Resectable	Surgery	Not applicable	Distal pancreatectomy
24	SNU-4771	M	73	Tissue	Adherent	48.3	Polygonal	Resectable	Chemotherapy	FOLFIRINOX (Partial response)	Not applicable
25	SNU-4866	M	67	Tissue	Adherent	85.7	Polygonal	Resectable	Surgery	Not applicable	Pancreaticoduodenectomy
26	SNU-5177	F	57	Tissue	Adherent	92.3	Polygonal	Borderline resectable	Chemotherapy	FOLFIRINOX (Partial response)	Not applicable

*Organoid-derived cell lines

### Genomic mutations of pancreatic cancer cell lines

**Fig. 2:**
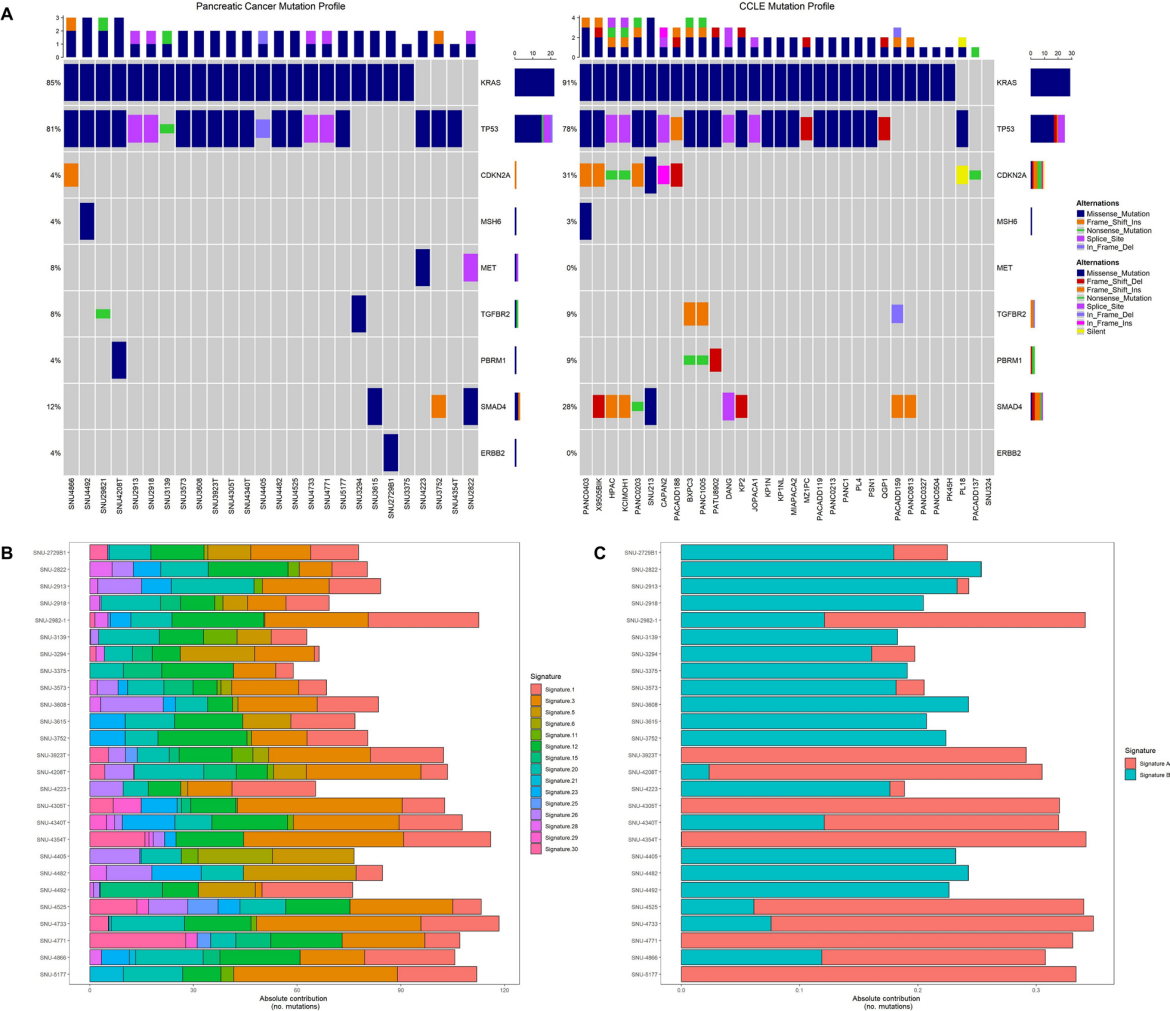
Genomic mutation profiles and contributions of signatures in pancreatic cancer cell lines **A** Overview of mutations in PDAC driver genes observed in our PCCLs (n = 26) compared with the mutational landscape in CCLE cell lines (n = 32). **B** Absolute contributions of mutational signatures identified across the PCCLs. **C** Absolute contributions of the two dominant signatures extracted from our cohort

To characterize the genetic features of the cell lines, whole exome sequencing was conducted. In PDAC, mutations in *KRAS*, *TP53*, *SMAD4*, and *CDKN2A* are the most prominent. [17] We selected nine driver genes including *KRAS*, *TP53* and *CDKN2A,* to provide an overview of the general mutational profiles of 26 PCCLs. We utilized the Clinvar database (https://www.ncbi.nlm.nih.gov/clinvar) to avoid misestimating the mutation frequency that could result from including germline or benign mutations. The clinical significance of the selected mutations was manually examined. Major mutations such as *KRAS*, *TP53*, *CDKN2A*, and *SMAD4* are summarized in Additional file 1: Table.S2A. All marked mutations used to calculate the frequency of mutated genes shown in Fig. 2A were previously reported as “Pathogenic”, “Likely Pathogenic”, or “Not reported” in the Clinvar database (Additional file 1: Table. S2A). The mutational frequencies of driver genes were compared between our cohort and 32 Cancer Cell Line Encyclopedia (CCLE) pancreatic cell lines (Fig. 2A). [18] The mutation frequencies of *KRAS* and *TP53* in our samples were similar to those in the CCLE pancreatic cancer cohort. Compared to CCLE cell lines, samples in our cohort contained fewer mutations in *CDKN2A* and *SMAD4*.

*KRAS*, the most commonly altered gene in pancreatic cancer, was mutated in 22 (85%) of the 26 cell lines in the present study. Numerous oncogenic *KRAS* alleles were identified, including G12D (59%), G12V (23%), and G12R (18%). Patients with wild-type *KRAS* often carry other oncogenic mutations, such as *SMAD4*, which may serve as candidates for small-molecule therapy. Among the *KRAS* wild-type cell lines, SNU-3752 harbored an oncogenic frameshift mutation in *SMAD4*. Additionally, SNU-4223 and SNU-4354T had oncogenic missense mutations in *TP53*.

Other commonly found genetic alterations included *TP53* and *SMAD4*. *TP53* mutations were found in 21 of the 26 cell lines which consisted of missense mutations, splice site mutations, non-sense mutations, and in-frame deletions. In addition, a *CDKN2A* mutation, known to inhibit the kinase activity of CDK4 and CDK6, [5] was detected in SNU-4866. *TGFBR2* mutations were found in SNU-2982-1 and SNU-3294. SNU-2729B1 harbored a missense mutation in *ERBB2*, SNU-4492 carried a frameshift mutation in *MSH6,* and SNU-4208T had a missense mutation in *PBRM1*.

We also examined the mutational signatures of PCCLs. The absolute contributions of various mutational signatures across the cell lines revealed a predominant contribution of Signature 1, Signature 3, and Signature 12 (Fig. 2B). Among the six substitution types, C > T mutations exhibited the highest relative contribution, particularly at CpG sites (Additional file 1: Fig.S2A, Table S2B). Figure 2C showed the absolute contributions of the two dominant signatures extracted from our cohort, which provided a clearer stratification of the cell lines. We analyzed the trinucleotide context of the mutations, grouped by substitution type (Additional file 1: Fig. S2B). C > T mutations showed enrichment in CpG dinucleotide contexts. These findings emphasized the diversity of mutational signatures within our cohort.

Taken together, our mutational analysis indicated that our pancreatic cancer cell lines presented the most frequently detected mutations in PDAC. However, due to the lack of normal samples in this study, it was challenging to identify pathogenic mutations. For that reason, we sought to overcome these challenges by excluding benign mutations and comparing the major somatic mutations of PDAC with those in the CCLE dataset.

### Transcriptomic profiling of cell lines classified two clusters that show distinct expression patterns

In our study, we applied consensus clustering using hierarchical clustering with Pearson distance to classify the samples. The results from consensus classification were used as a baseline for comparison and validation of subsequent analyses. Despite a few differences, the Consensus Cluster (CC) results demonstrated strong concordance with the findings from Principal Component Analysis (PCA), VST heatmap, Gene Set Variation Analysis (GSVA), and Weighted Correlation Network Analysis (WGCNA). To quantitatively assess these relationships, we calculated the Jaccard similarity scores among the clusters derived from the five methods (Additional file 1: Fig. S3A, Table S3). These similarity scores revealed high correlations with the CC results, validating the use of consensus clustering as an effective and reliable method for unsupervised classification in our cohort. Four cell lines (SNU-2822, SNU-2982-1, SNU-4771, and SNU-5177) were excluded from RNA sequencing analysis.

**Fig. 3:**
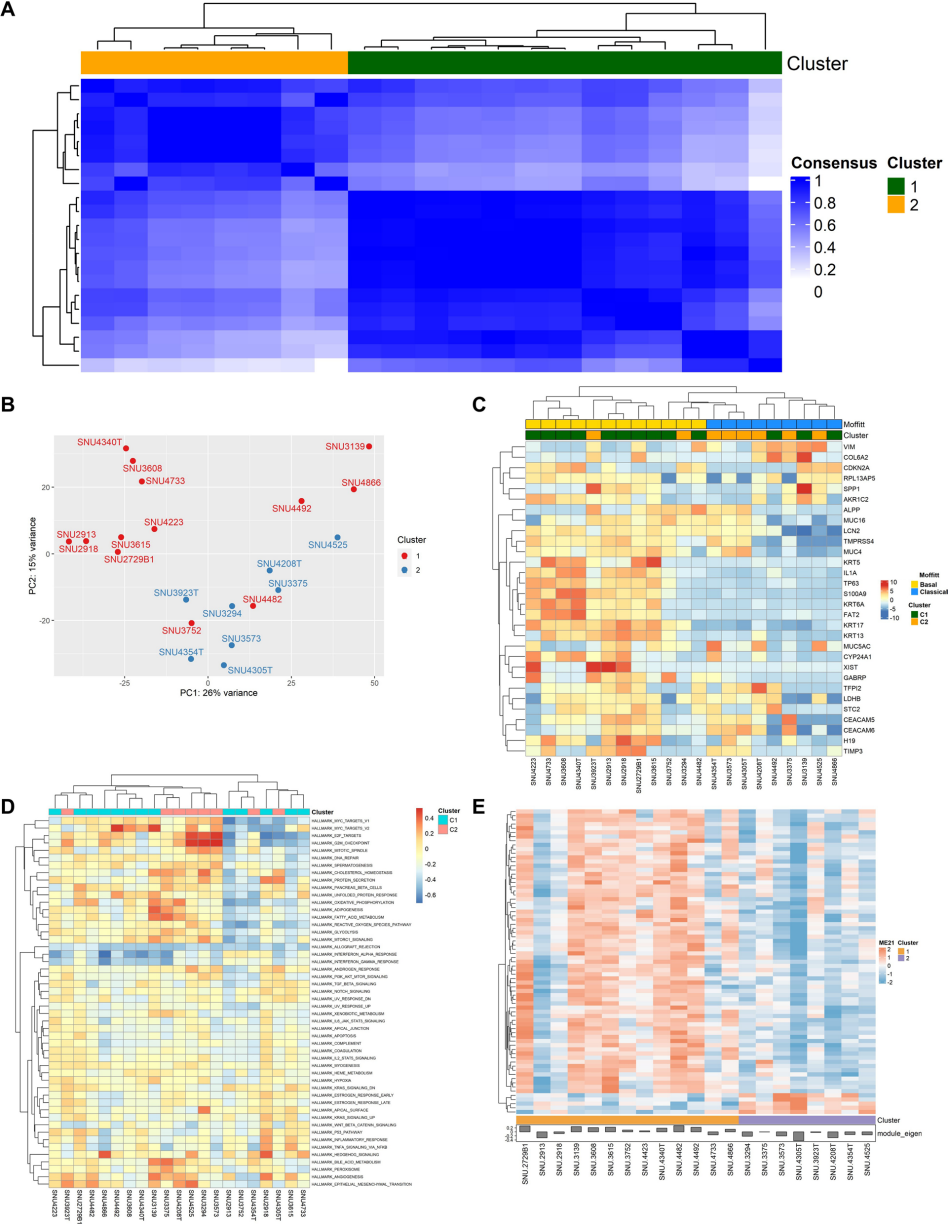
Transcriptomic analysis of PCCLs. **A** Consensus clustering of mRNA profiles in PCCLs for the optimal number of clusters at k = 2. **B** Principal component analysis (PCA) identified two major mRNA clusters. **C** Heatmap of relative VST-transformed values across samples. Selected 30 genes with the highest variance across samples. With Moffitt’s classification, C1 shares common expressions with “Basal-like” type and C2 with “Classical” type. **D** Heatmap displayed GSVA enrichment scores for hallmark pathways across pancreatic cancer cell lines highlighting differentially expressed pathways between the two clusters of PCCLs. **E** Heatmap identified gene co-expression modules associated with the two clusters

The PCCLs were divided into two clusters classified by consensus clustering (Fig. 3A). The ConsensusClusterPlus R package was used to determine clusters using expression levels of associated genes, employing the default settings. [15] Based on these analyses, k = 3 was identified as the theoretical optimal value according to the CDF Plot and the delta area Plot (Additional file 1: Fig. S3B-C). However, we found that using k = 3 resulted in overfitting and decreased interpretability, as the clustering pattern indicated that only one sample was annotated as a separate cluster.

For our study, we selected k = 2, which provided a balance between clustering comprehensibility and interpretability. This choice also avoided overfitting and ensured meaningful clusters for downstream analyses. Although higher k values can provide more detailed cluster resolution, they often lead to more complex results and reduced clarity in interpretation. Given our study’s objectives, choosing k = 2 offered a more practical and molecularly meaningful framework for understanding our cohort.

The program optimally separated our cell lines into two groups. Four of the cell lines (SNU-4305T, SNU-3923T, SNU-4208T, and SNU-4354T), derived from organoids were in C2, whereas one (SNU-4340T) was classified into C1.

Distinct molecular characteristics can be observed in cell lines obtained from different cancer subtypes. Principal component analysis (PCA) was used to identify the major genetic factors dividing PDAC cell line samples based on mRNA expression (Fig. 3B). As shown in Fig. 3B, C1 and C2 were highly separated along PC2 which accounted for 15% of the variance. Cell lines derived from organoids clustered in C2, except for SNU-4340T. PCA of the dataset revealed that the first principal component (PC1) accounted for 24.62% of the total variance, followed by PC2 at 15.01% (Additional file 1: TableS4). The scree plot demonstrated the variance explained by each principal component (PC) in the PCA (Additional file 1: Fig. S3D). Together, PC1 and PC2 captured approximately 40% of the total variance. These findings suggest that the first two PCs sufficiently represent the major patterns in the dataset. Subsequently, we further analyzed PC2 by pinpointing the top 15% (n = 4153 genes) among the total PC2 loadings.

The top 15% (n = 4153 genes) of PC2 loadings (Additional file 1: Fig. S3E) were extracted using the PCA tools R package and compared with the DESeq2 results for C1 and C2. Functional enrichment analysis was performed on the differentially expressed genes to determine the pathways enriched in each cluster. Using Moffitt’s classification method, C1 displayed traits similar to the “Basal-like” type, while C2 aligned with the “Classical” subtype. The heatmap of VST-transformed values highlighted 30 genes with the highest variance across samples (Fig. 3C). Cell lines in C1 exhibited higher gene expression compared to those in C2, where most cell lines had low gene expression, except for SNU-3923T, which showed notable exceptions in XIST and SPP1 expression. In comparison to other methods, GSVA proved highly practical for detecting small pathway variations across a sample population [19]. The calculated pathway-level scores revealed differences in gene activity within a gene set compared to those outside the gene set. Cell cycle regulation pathways appeared to cluster together and exhibited similar expression patterns (e.g., HALLMARK_MYC_TARGETS_V1, HALLMARK_MYC_TARGETS_V2, HALLMARK_E2F_TARGETS, HALLMARK_G2M_CHECKPOINT, HALLMARK_MITOTIC_SPINDLE) (Fig. 3D). These results demonstrated clear expression patterns distinguishing C1 and C2.

**Fig. 4:**
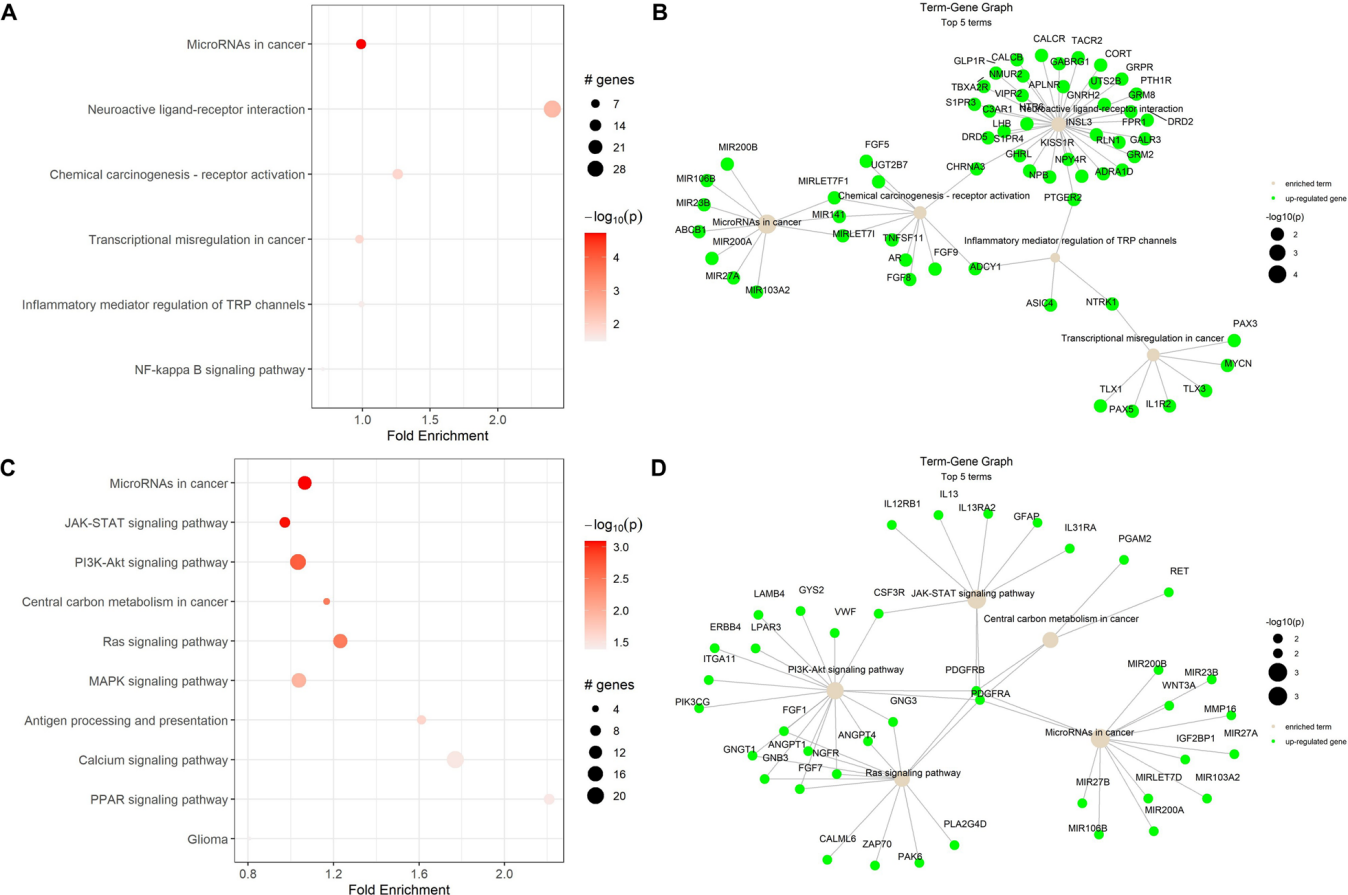
Active-subnetwork oriented enrichment analysis of C1 and C2 using the KEGG database. **A** Enrichment chart for C1: Dot plot showing the top six enriched terms identified in C1 using KEGG pathway analysis. **B** Term-Gene graph for C1: A network visualization showing significant genes involved in the top five enriched terms in C1. The graph illustrates overlaps between enriched terms by identifying shared genes. **C** Enrichment chart for C2: Dot plot showing the top ten enriched terms identified in C2. **D** Term-Gene graph for C2: A network representation of the top five enriched terms

To determine significant pathways expressed in each cluster, we used the pathfindR R package. For C1, the number of genes provided in the input was 4153 and the number of genes remaining after p-value filtering was 1772. PathfindR could not handle p-values < 1e−13, so 851 genes (48.02%) were excluded from interaction analysis. The final number of genes in the input was 921 for C1. The results identified six enriched terms by annotating the involved genes and visualizing the enriched terms (Fig. 4A). The term-gene graph for C1 showed associations of miRNA genes such as *MIRLET7F1*, *MIR141*, and *MIRLET7I* with chemical carcinogenesis-receptor activation pathways (Fig. 4B). For C2, the number of genes remaining after p-value filtering was 2,014, and the final number of genes in the input was 1,112. This analysis identified ten enriched terms (Fig. 4C). Notable terms included MicroRNAs in cancer, JAK-STAT signaling pathway, PI3K-Akt signaling pathway, Central carbon metabolism in cancer, and Ras signaling pathway. Furthermore, the term-gene graph for C2 revealed interrelations between genes *PDGFRB* and *PDGFRA* with the five pathways described above (Fig. 4D).

WGCNA was applied to determine gene co-expression modules associated with the two clusters using the R package WGCNA [20]. We selected a power parameter based on the scale independence plot. The authors of WGCNA recommend using a power parameter with a signed R^2^ value above 0.80 to ensure reliable results. Module 21 showed elevated gene expression in C1 compared to C2 (Fig. 3E).

We aimed to determine whether any Gene Ontology (GO) molecular functions were enriched among overexpressed genes differentiating the two pancreatic cancer clusters. The R Package DESeq2 was used to perform differential analysis on RNA sequencing read count data. From the DESeq2 results of each cluster, we selected the top 15% results with a p-value < 0.05. GO overrepresentation analysis of C1 and C2 was then conducted using the R package ClusterProfiler. GO biological pathways overrepresented in C1 included the adenylate cyclase-modulating G protein-coupled receptor signaling pathway, cell–cell adhesion via plasma-membrane adhesion molecules, and sodium ion transport (Additional file 1: Fig. S4A, B, C). Pathways overrepresented in C2 included muscle contraction, sodium ion transport and potassium ion transport (Additional file 1: Fig. S5A, B, C).

Gene set enrichment analysis (GSEA) of differentially expressed genes in C1 and C2 was performed using ClusterProfiler with GO terms and the KEGG database. Our analysis revealed multiple activated processes in C1, including the phosphate-containing compound metabolic process and transmembrane receptor protein serine/threonine kinase signaling pathway (Additional file 1: Fig. S4D). In contrast, C2 exhibited enrichment in the nucleic acid metabolic process and DNA-binding transcription factor activity (Additional file 1: Fig. S5D). We also used the same package to visualize enriched KEGG pathways associated with genes of interest in C1 and C2. C1 was enriched in pathways such as JAK-STAT signaling pathway and cytokine-cytokine receptor interaction (Additional file 1: Fig. S4E). C2 was enriched in pathways related to Herpes simplex virus 1 infection and Salmonella infection (Additional file 1: Fig. S5E).

Uncovering the molecular characteristics of pancreatic cancer subtypes is crucial because the biological activities dominating PDAC oncogenesis could provide a foundation for future therapeutic strategies.

### Drug screening of cell lines reveals sensitivities to a range of therapeutic agents

All 26 established cell lines were exposed to 18 anti-cancer drugs, including those currently used in the treatment of PDAC (Additional file 1: Fig. S6A). Our drug screening library was partially informed by the the clinical history of patients’ chemotherapy regimens. Most of the patients received FOLFIRINOX or gemcitabine after surgery. Using this information, we included fluorouracil, irinotecan, and gemcitabine in the screening library.

**Fig. 5:**
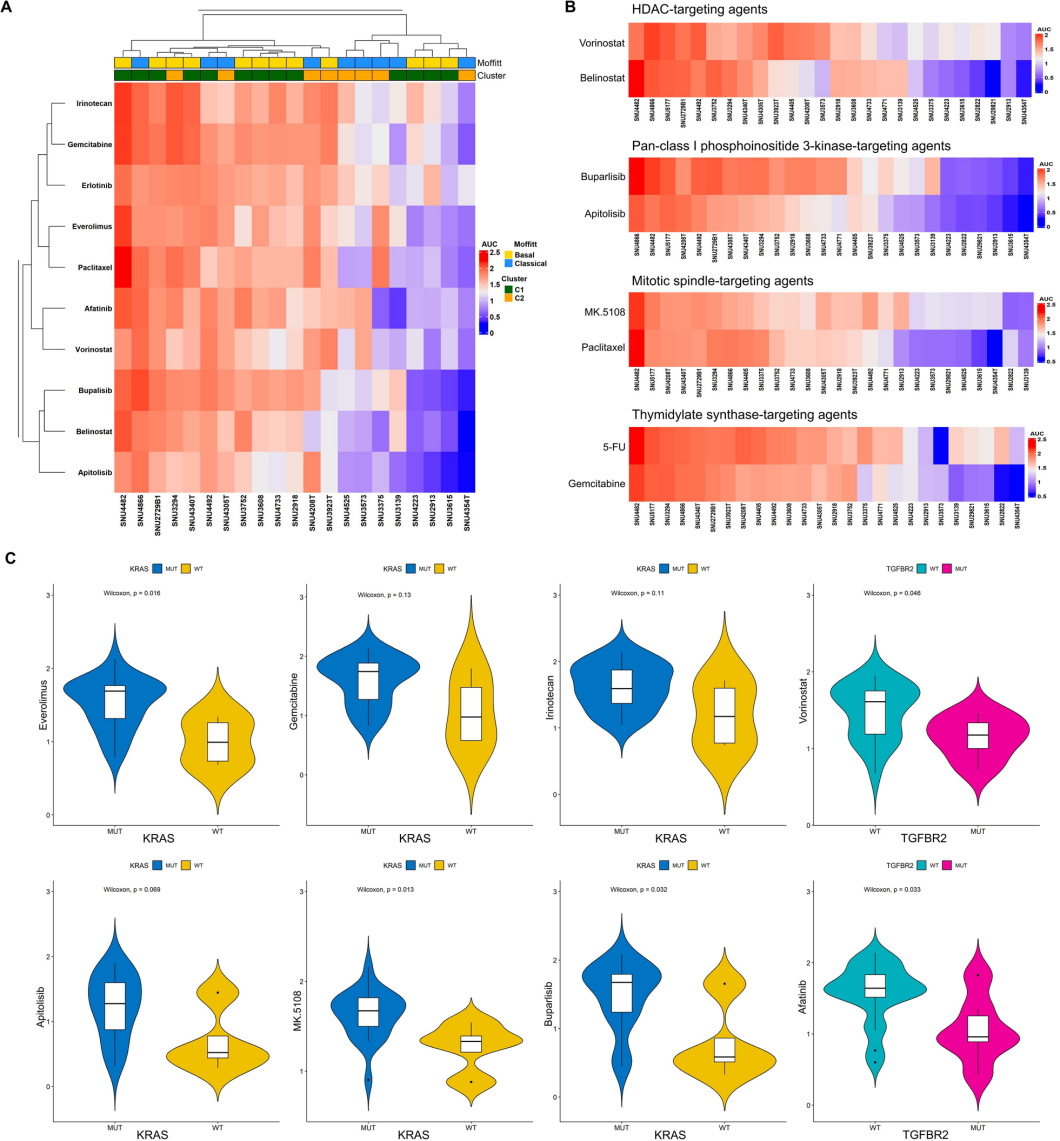
Pancreatic cancer cell lines reveal heterogenous drug responses caused by molecular diversity **A** Heatmap of PCCLs exhibited distribution of 10 compounds according to their molecular characteristics. The cell lines and drugs were k-means clustered based on AUC values across the drug panel. High values indicating resistance are depicted in red, and low values indicating sensitivity are in blue. **B** Results of PCCLs to compounds targeting the same biological process or pathway highlighting similar responses observed among the different compounds. **C** Violin plot of the correlation status between KRAS and AUC of several drugs. Violin plot of the correlation status between TGFBR2 and two anticancer drugs

The area under the curve (AUC) was used to assess the sensitivities of PCCLs to these compounds. Different sensitivity patterns were observed in a subset of cell lines used for transcriptomic analysis (Fig. 5A). In our cohort, C2 was more sensitive to anticancer drugs than C1. In particular, sensitivity was evident for Buparlisib and Apitolisib, which specifically inhibit class I PIK3 in the PI3K/Akt signaling pathway. This finding was consistent with our transcriptomic analysis results, where C2 showed overexpression of the PI3K-Akt signaling pathway. Anticancer drugs targeting similar molecular pathways showed comparable responses in PCCLs (Fig. 5B). Vorinostat and Belinostat both target histone deacetylases (HDAC); Buparlisib and Apitolisib target pan-class I phosphoinositide 3-kinase (p110α/β/δ/γ); MK-5108 and Paclitaxel inhibit mitotic spindle assembly; and 5-FU and gemcitabine target thymidylate synthase.

To validate the correlation between mutation status and drug response in our sample cohort, we performed both a multivariate analysis of variance (MANOVA) and a Wilcoxon rank test. Results from MANOVA identified associations between *KRAS* and several drugs, including everolimus, gemcitabine, irinotecan, apitolisib, MK-5108, and buparlisib. In addition, a strong interrelation was observed between vorinostat and *TGFBR2,* as well as between afatinib and *TGFBR2*. We used the Wilcoxon rank test to further evaluate the relationship between gene mutations and drug response. *KRAS* WT showed increased sensitivity to agents compared to cell lines with a *KRAS* mutation (Fig. 5C). Our dataset included four *KRAS*-WT cell lines, providing a unique opportunity to statistically compare drug responses based on *KRAS* mutational status. This underscored the distinctiveness and value of our dataset for exploring *KRAS*-dependent drug sensitivities. In contrast, for *TGFBR2*, mutation of the gene was associated with higher drug intolerance. The results indicated that sensitivities to chemotherapeutic agents differed significantly depending on the mutational status of *KRAS* and *TGFBR2*.

As mentioned previously, *KRAS* is the major oncogenic driver of PDAC. The most common mutation site in *KRAS* occurs at codon 12, predominantly in the G12D mutation [21]. Numerous studies have targeted mutant *KRAS* in efforts to develop inhibitors for the most recurrent *KRAS* G12D mutation. Recently, MRTX1133, a noncovalent, selective inhibitor of *KRAS* G12D, was discovered and proven to be effective in a *KRAS* G12D mutant xenograft mouse tumor model [22]. Since 22 (85%) of the 26 cell lines in our study contained a *KRAS* mutation, we sought to evaluate the efficacy of MRTX1133. As expected, cell lines with *KRAS* G12D were sensitive to the inhibitor. Furthermore, cell lines with *KRAS* G12V mutations also demonstrated sensitivity to MRTX1133, which can be explained by recent studies showing the activity of MRTX1133 on *KRAS* G12V [23]. However, further studies are essential to confirm the potency of MRTX1133 (Additional file 1: Fig. S6B). If MRTX1133 advances to clinical trials and demonstrates successful anticancer effects, the impact of targeting *KRAS* G12D mutations will be enhanced.

Of the patients analyzed, 10 received FOLFIRINOX or 5-FU, categorized as a 5-FU based regimen, while 2 were treated with a gemcitabine-based regimen (Additional file 1: TableS5). Treatment responses were classified as Partial Response (PR; Sensitive) > Stable Disease (SD) > Progressive Disease (PD; Resistant). To evaluate the translational applicability of our findings, we compared the AUC values for each regimen with their corresponding clinical treatment responses. For patients treated with the 5-FU based regimen, we divided them into PR and SD/PD groups and compared their average AUC values. Although the PR group exhibited a lower AUC value, the difference was small. For the gemcitabine-based regimen, the patient with SD had a lower AUC value compared to the patient with PD, which aligns with our in vitro results. However, due to the limited number of samples, drawing statistically significant conclusions is challenging. Expanding the dataset to include more clinical data would greatly improve the translational relevance of these findings.

### Protein expression profiles through western blot analysis

**Fig. 6:**
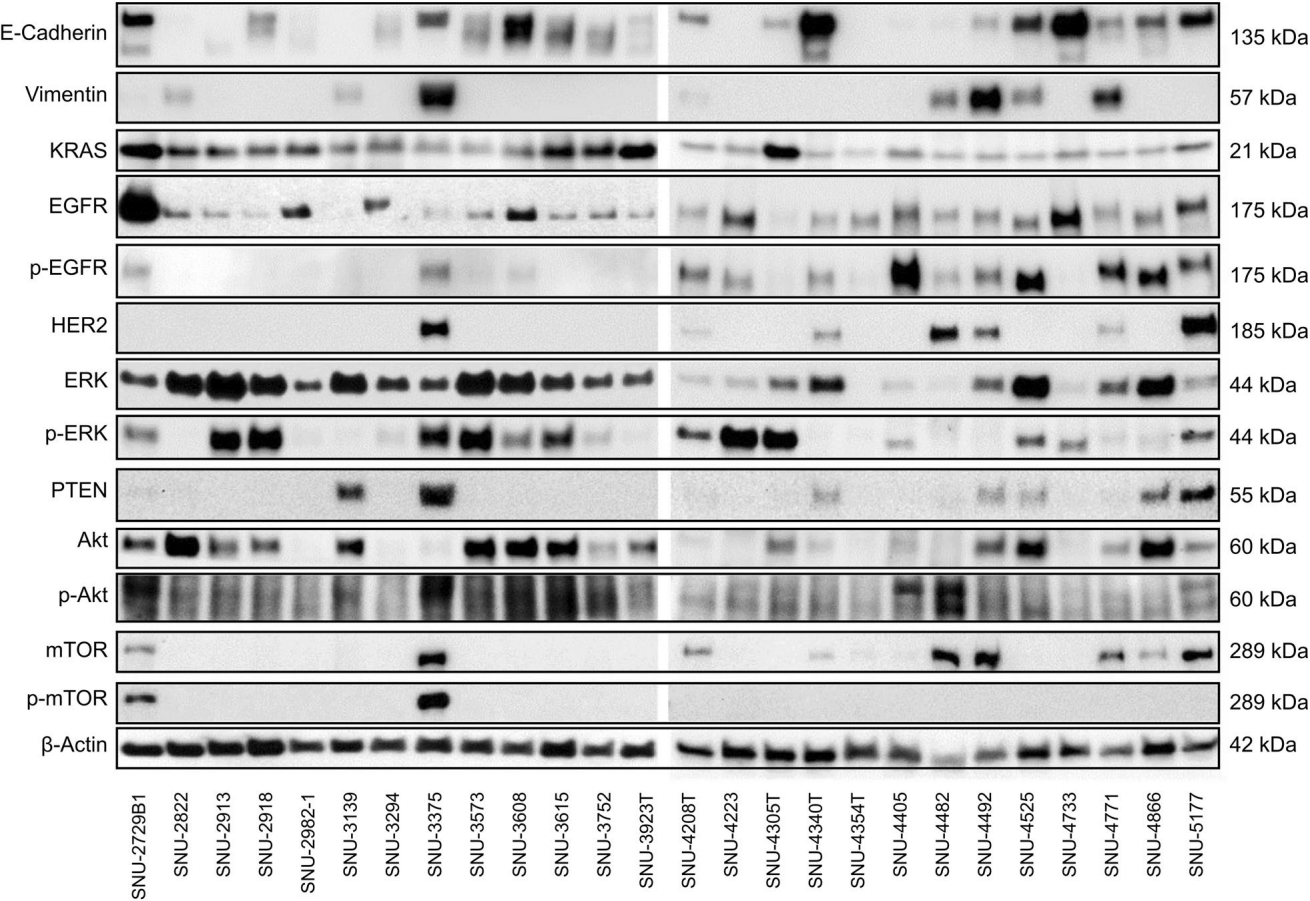
Western blot analysis of protein expression in 26 pancreatic cancer cell lines. Western blot analysis showing the expression levels of key markers and signaling proteins across cell lines

Protein expression was confirmed by western blotting. PTEN, Akt, p-Akt, mTOR, and p-mTOR levels were detected to assess the activity of the Akt/mTOR pathway. KRAS, EGFR, p-EGFR, HER2, ERK, and p-ERK levels were detected to evaluate the RAS-MEK-ERF pathway (Fig. 6). Western blot quantification of the cell lines is shown in Additional file 1: Table S6. Differences in protein expression between cell lines indicated tumor heterogeneity.

In terms of pathway activity, highly activated EGFR cell lines tended to exhibit elevated expression levels of the downstream cascade component p-ERK, although these results were inconsistent. Similarly, the activity of p-mTOR appeared to be induced by p-Akt, but this finding was also discordant. Representatively, the expression levels of p-EGFR and HER2 in SNU-4340T, SNU-4492, SNU-4866, and SNU-2822 did not align with the activity of p-ERK and p-Akt.

Our KRAS wild-type cell lines demonstrated lower activity of signaling proteins compared to other subgroups. This was evident in the reduced activation of p-EGFR, HER2, and p-mTOR by p-Akt.

Subgroups with *KRAS*-mutated cell lines exhibited diverse inter-tumor heterogeneities, representing the complexity of pancreatic cancer. Among them, PTEN, an important tumor suppressor gene of the Akt/mTOR pathway, was expressed in four cell lines (SNU-3139, SNU-3375, SNU-4866, and SNU-5177) that exhibited significant resistance to everolimus, which targets the mTOR pathway.

Interestingly, cell lines overexpressing HER2 were more frequently mutated than previous studies. The proportion was about 26% which was 7 out of 26 cell lines resulting in a significantly higher percentage than the reported 2% [24]. This subgroup exhibited higher activation of signaling proteins than the previous two groups. Since it expressed p-EGFR and HER2, it showed higher activity of signal transduction proteins p-ERK and p-Akt, as well as elevated basal expression levels of mTOR. These characteristics are believed to contribute to the resistance observed in some of these cell lines to anticancer drugs.

## Discussion

Over the years, numerous studies have been conducted to determine the causes of PDAC development. PDAC remains a major clinical challenge due to its poor prognosis and lack of response to therapies for this lethal disease.

In this study, a biobank of 26 pancreatic cancer cell lines derived from tissue and organoids was used for genetic characterization. Our cohort harbored commonly identified genetic mutations, such as *KRAS* and *TP53*, in concordance with the mutational profiles of external datasets consisting of PCCLs (e.g., CCLE). The high prevalence of *KRAS* and *TP53* mutations was anticipated, as these are the most commonly identified genomic mutations in PDAC. These results showed that our cell lines largely maintain the common oncogenic drivers associated with PDAC.

We classified PCCLs into two clusters using transcriptomic data. Consensus clustering of 22 cell lines divided the samples into two distinct clusters, which were then used for further analysis. Despite the small sample size, it was notable that our cohort clearly divided into two clusters. While we attempted to sort our cell lines according to the classification presented by Bailey, they did not correspond to the Bailey subtypes (Additional file 1: Fig. S7), likely due to the inherent heterogeneity among cell lines and the fact that Bailey subtypes are based on gene expression profiles derived from patient tumor tissues. The two clusters C1 and C2, matched Moffitt’s classification of “Classical” and “Basal-like” subtypes and showed similar pathway expressions as previously reported. C1 exhibited upregulated pathways involved in inflammation, such as the NF-kappa B signaling pathway, while C2 showed upregulated pathways related to PI3K-Akt signaling and cell adhesion. Heatmap results from GSVA revealed that cell cycle regulation pathways clustered together. A previous study indicated that the high frequency of cell proliferation-related gene sets, including the G2M checkpoint pathway, was significantly associated with *KRAS* mutations and worse patient survival [25]. Our transcriptomic analysis also identified associations between *PDGFRa* and *PDGFRb* and significant pathways expressed in C2, highlighting their frequent presence in highly aggressive tumors, including PDAC [26]. Verifying heterogeneous populations of cell lines with the limited gene sets from Moffitt’s classification is challenging; hence, we analyzed total transcriptomic patterns from mRNA data, which explains the few discrepancies observed. These findings demonstrate that PCCLs can be clustered with mRNA data alone, suggesting that transcriptomic data depth is a powerful tool for identifying oncogenic drivers with potential therapeutic significance. Future studies with larger datasets are needed to validate the predictive potential of patient-derived PCCLS for clinical applications.

To identify effective therapies for pancreatic cancer, drug screening was performed using a panel of 18 compounds. Our results support the hypothesis that therapies targeting the same molecular pathway may be effective for specific subsets of patients, emphasizing the importance of a personalized approach in identifying effective treatments. Studies showed that inhibiting PI3K/Akt pathway improves the chemosensitivity of PDAC cell lines in vitro and in vivo [27]. Because PI3Ks and AKTs are considered essential proteins and promising therapeutic targets, inhibitors of these pathways have shown optimistic results in PDAC treatment [28]. Given the low treatment responses observed in patients with PDAC, targeting this pathway could offer promising outcomes for patients with PI3K and AKT. To assess the sensitivity profiles of different cell line subgroups, we combined drug sensitivity data and identified drugs that displayed varying efficacy. Using MANOVA and the Wilcoxon test, we confirmed genomic mutations associated with differential drug sensitivity. These analyses revealed molecular characteristics correlated with drug response, providing potentially effective approaches for precision treatments in PDAC. Notably, C1, which exhibited characteristics to the “Basal-like” subtype, was less responsive to drugs than C2. To improve subtype categorization and address the observed dissimilarities, including additional genes in the analysis would be beneficial. Our results were consistent with previously established gene-drug databases, confirming that clinically used drugs, such as Gemcitabine and Everolimus repeatedly correlated with *KRAS* mutation status. [29] These findings suggest that cell line models are effective tools for predicting drug responses, particularly for targeted therapies.

Focus on *KRAS*-targeted therapies has been increasing due to the high frequency of *KRAS* mutations in PDAC patients. Despite this, current strategies to directly target mutant *KRAS* proteins have been unsuccessful due to their high affinity for GTP and/or GDP [11]. We used MRTX1133 to confirm the validity of its function. Our results indicate that the inhibitor does target *KRAS* G12D mutation. However, further research is required to clarify the mechanisms underlying this sensitivity.

We analyzed whether cell lines in clusters C1 and C2 correlated with growth rates (Additional file 1: TableS7A). The results indicated that the average growth rates for both clusters were similar, with C2 exhibiting a slightly higher rate. However, a two-tailed test yielded a p-value of 0.87, suggesting that the observed difference in means was minimal. Additionally, the p-values indicate no statistically significant difference between the growth rates of C1 and C2. These findings suggest that growth rate is not a major contributor to the clustering observed in our dataset. We also performed a Pearson correlation analysis to assess the relationship between doubling times and the response to all drugs used (Additional file 1: TableS7B). The results indicated that for most drugs the correlations were weak, suggesting no statistically significant association. However, Trametinib (r = 0.462, p = 0.017) and Cyclopamine (r = 0.396, p = 0.045) showed moderate correlations with statistically significant p-values, which suggested that faster-growing cell lines may exhibit greater sensitivity to these drugs (Additional file 1: FigureS8).

Cancer cell lines are invaluable tools for drug sensitivity prediction, a crucial component of precision oncology. PCCLs retain key molecular characteristics and genomic mutations of PDAC, allowing researchers to gain significant insights into cancer biology. There are 82 publicly available human pancreatic cancer cell lines that are well-characterized through repositories such as ATCC, DSMZ, RCB, JCRB, ECACC, and KCLB. In addition, CCLE provides datasets of 32 PCCLs. However, the current datasets are scarce in number to fully capture the interpatient heterogeneity in PDAC.

We have previously established and characterized 14 PCCLs in our lab. [30, 31] With the addition of 26 newly developed PCCLs, we used advanced sequencing techniques and expanded the drug screening library to analyze correlations between genomic, transcriptomic, and drug response data. Nonetheless, defining how well patient-derived cell lines resemble actual PDAC tumors remains challenging. Despite these limitations, our findings underscore the importance of larger PCCL datasets for achieving a more comprehensive understanding of the molecular mechanisms underlying PDAC. Such insights are important for improving survival rates and quality of life for pancreatic cancer patients. By integrating genomic, transcriptomic, and drug sensitivity data, PCCLs have the potential to uncover future therapeutic strategies through molecular subtype classification.

## Supplementary Information

Additional file 1. Figure S1. Mycoplasma results with the 16 s-rRNA-gene based polymerase chain reaction. PCR was performed to confirm the infection of mycoplasma. All cell lines were free of mycoplasma contamination

Additional file 2. Figure S2. Signature contributions in PCCLs.Relative contributions of point mutation types were estimated in cell lines, with each mutation type represented by distinct colors.Relative contributions of Signature A and Signature B shown across trinucleotide contexts

Additional file 3. Figure S3.Heatmap illustrating the Jaccard similarity scores between clustering methods, including consensus clustering, PCA, GSVA, WGCNA, and VST heatmap.CDF curves for different cluster numbersto assess cluster stability across PCCLs.Plot showing the relative change in area under the CDF curve.Scree plot of percentage of variance explained by each principal component.Loadings of genes across the first five principal components

Additional file 4. Figure S4. Differential gene expression analysis of C1.GO biological pathways overrepresented in C1 showed adenylate cyclase-modulating G protein-coupled receptor signaling pathway, cell–cell adhesion via plasma-membrane adhesion molecules and sodium ion transport.An enrichment map visualizing the connections between enriched GO terms in the C1 cluster.GSEA dot plot displayed activated and suppressed pathways for C1.Enriched pathways in C1 identified through KEGG pathway analysis

Additional file 5. Figure S5. Differential gene expression analysis of C2.GO biological pathways overrepresented in C2 showed muscle contraction, sodium ion transport and potassium ion transport.An enrichment map visualizing the connections between enriched GO terms in the C2 cluster.GSEA dot plot displayed activated and suppressed pathways for C2.Enriched pathways in C2 using KEGG pathways

Additional file 6. Figure S6. Drug sensitivity profiling and MRTX1133 response in PCCLs.AUC values for 18 anti-cancer drugs tested across PCCLs. Higher sensitivity is represented in blue, while lower sensitivity is represented in red.Viability curves of cell lines treated with MRTX1133 stratified by KRAS mutations status. Relative cell viability is plotted against the logarithm of drug concentration

Additional file 7. Figure S7. Heatmap of gene expression in PCCLs categorized by Bailey subtypes

Additional file 8. Figure S8.Correlation between doubling time and Cyclopamine sensitivity, represented by the AUC. A positive correlation was observed.Correlation between doubling time and Trametinib sensitivity, represented by the AUC

Additional file 9. Table S1. DNA fingerprinting results using 15 STR loci and the Amelogenin marker

Additional file 10. Table S2.Driver mutation profiles in PCCLs detected by whole exome sequencing.Point mutation type occurrences

Additional file 11. Table S3. Clustering of pancreatic cancer cell lines based on transcriptomic analysis

Additional file 12. Table S4. Variance contribution of principal components

Additional file 13. Table S5. AUC values for Gemcitabine and 5-FU in cell lines stratified by patient clinical regimens and responses

Additional file 14. Table S6. Quantification of protein expression by Western blot in 26 pancreatic cancer cell lines

Additional file 15. Table S7.Doubling times of C1 and C2 and statistical comparison using two-sample t-test.Pearson correlation analysis between doubling times and drug responses in cell lines

## Data Availability

The datasets during and/ or analyzed during the current study will be available from the corresponding author on reasonable request.
