# Supplementary figures and images for: Comprehensive molecular analysis of 26 newly established human pancreatic ductal adenocarcinoma cell lines reveals two clusters with variating drug sensitivities
Source: Cancer Cell Int. 2025 Feb 19;25:53. doi: 10.1186/s12935-025-03671-8 (PMC11837577; doi:10.1186/s12935-025-03671-8)

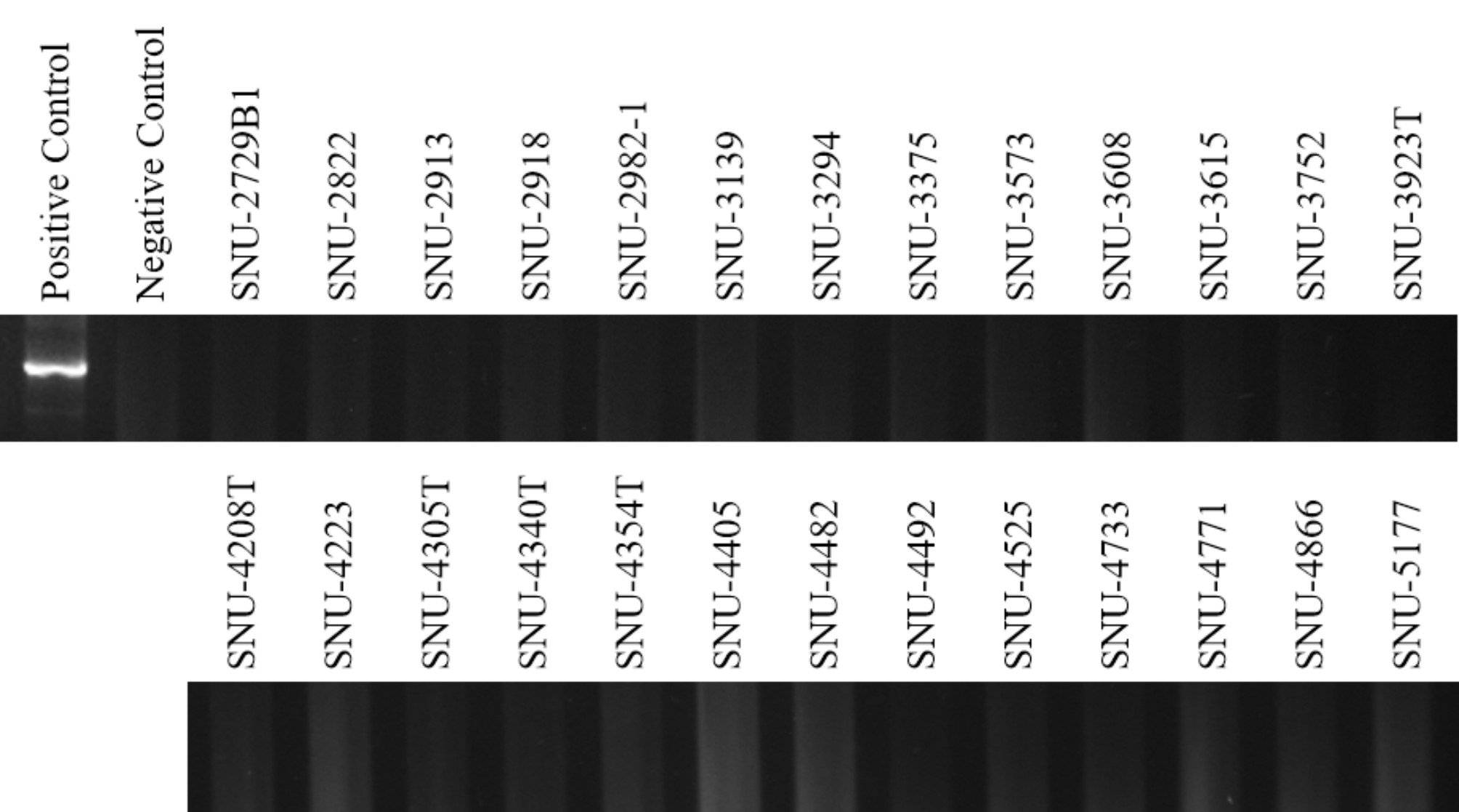

Supplement: Supplementary file 1 — Additional file 1. Figure S1. Mycoplasma results with the 16 s-rRNA-gene based polymerase chain reaction. PCR was performed to confirm the infection of mycoplasma. All cell lines were free of mycoplasma contamination [file 12935_2025_3671_MOESM1_ESM.jpg]

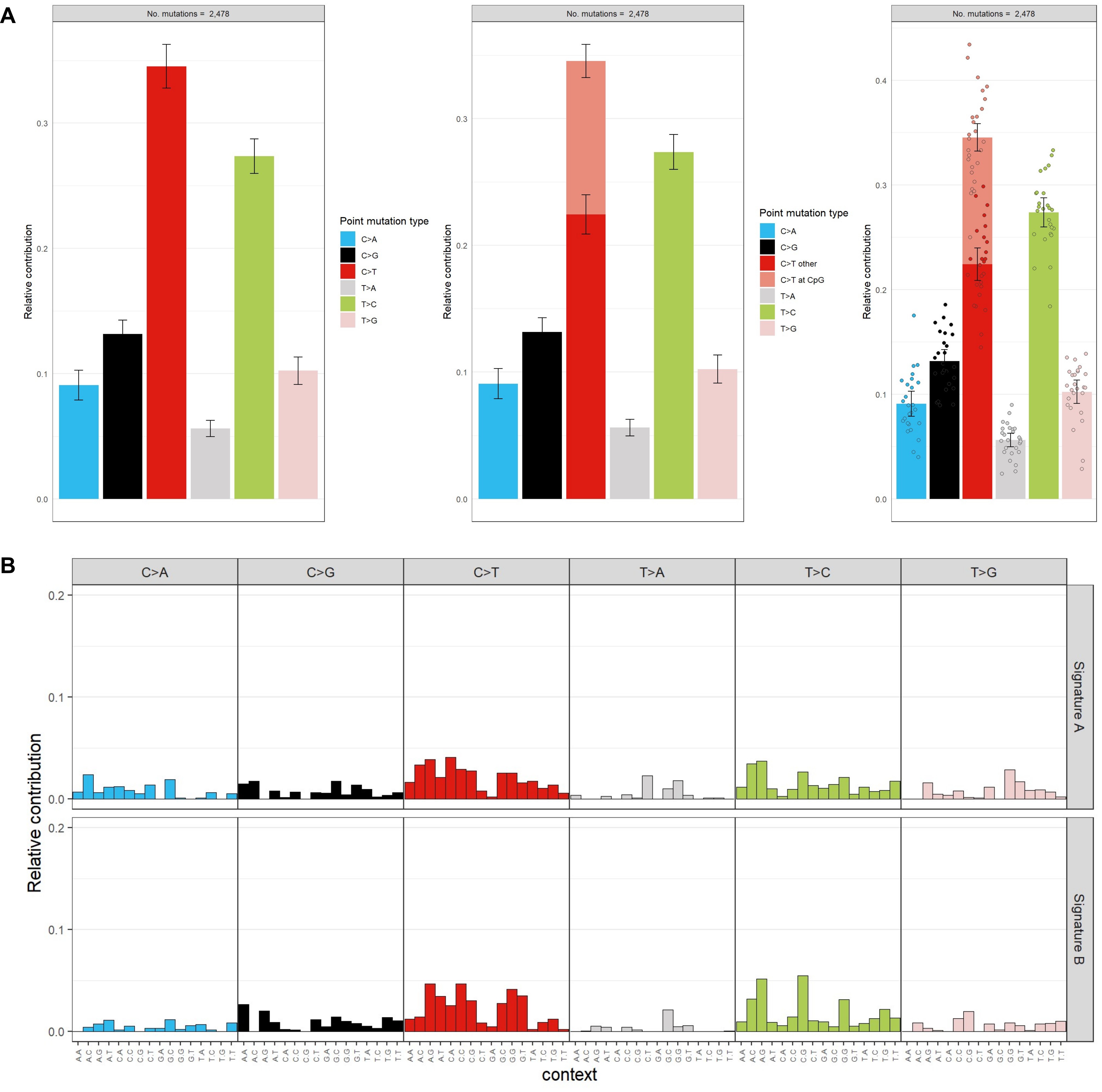

Supplement: Supplementary file 2 — Additional file 2. Figure S2. Signature contributions in PCCLs.Relative contributions of point mutation types were estimated in cell lines, with each mutation type represented by distinct colors.Relative contributions of Signature A and Signature B shown across trinucleotide contexts [file 12935_2025_3671_MOESM2_ESM.jpg]

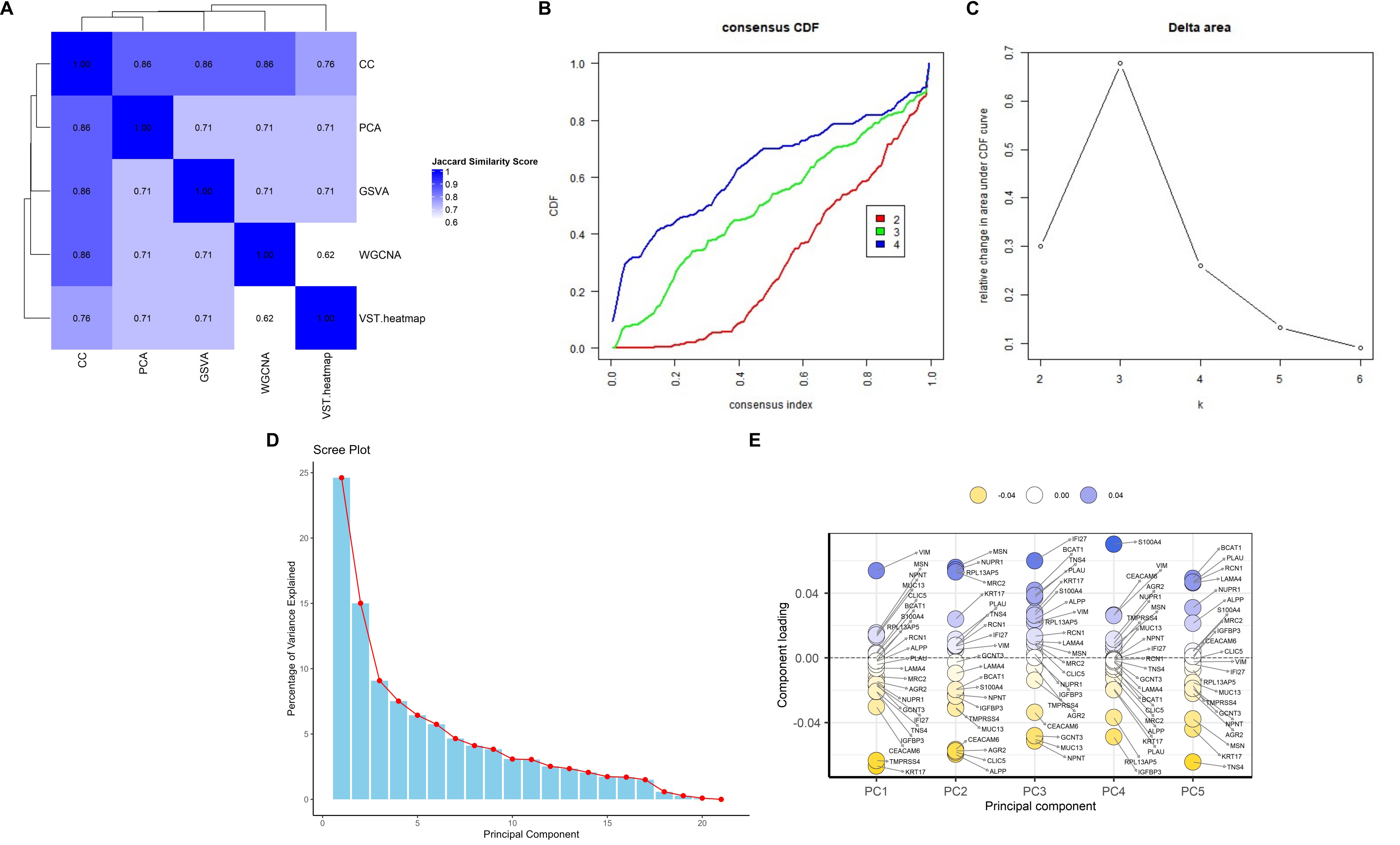

Supplement: Supplementary file 3 — Additional file 3. Figure S3.Heatmap illustrating the Jaccard similarity scores between clustering methods, including consensus clustering, PCA, GSVA, WGCNA, and VST heatmap.CDF curves for different cluster numbersto assess cluster stability across PCCLs.Plot showing the relative change in area under the CDF curve.Scree plot of percentage of variance explained by each principal component.Loadings of genes across the first five principal components [file 12935_2025_3671_MOESM3_ESM.jpg]

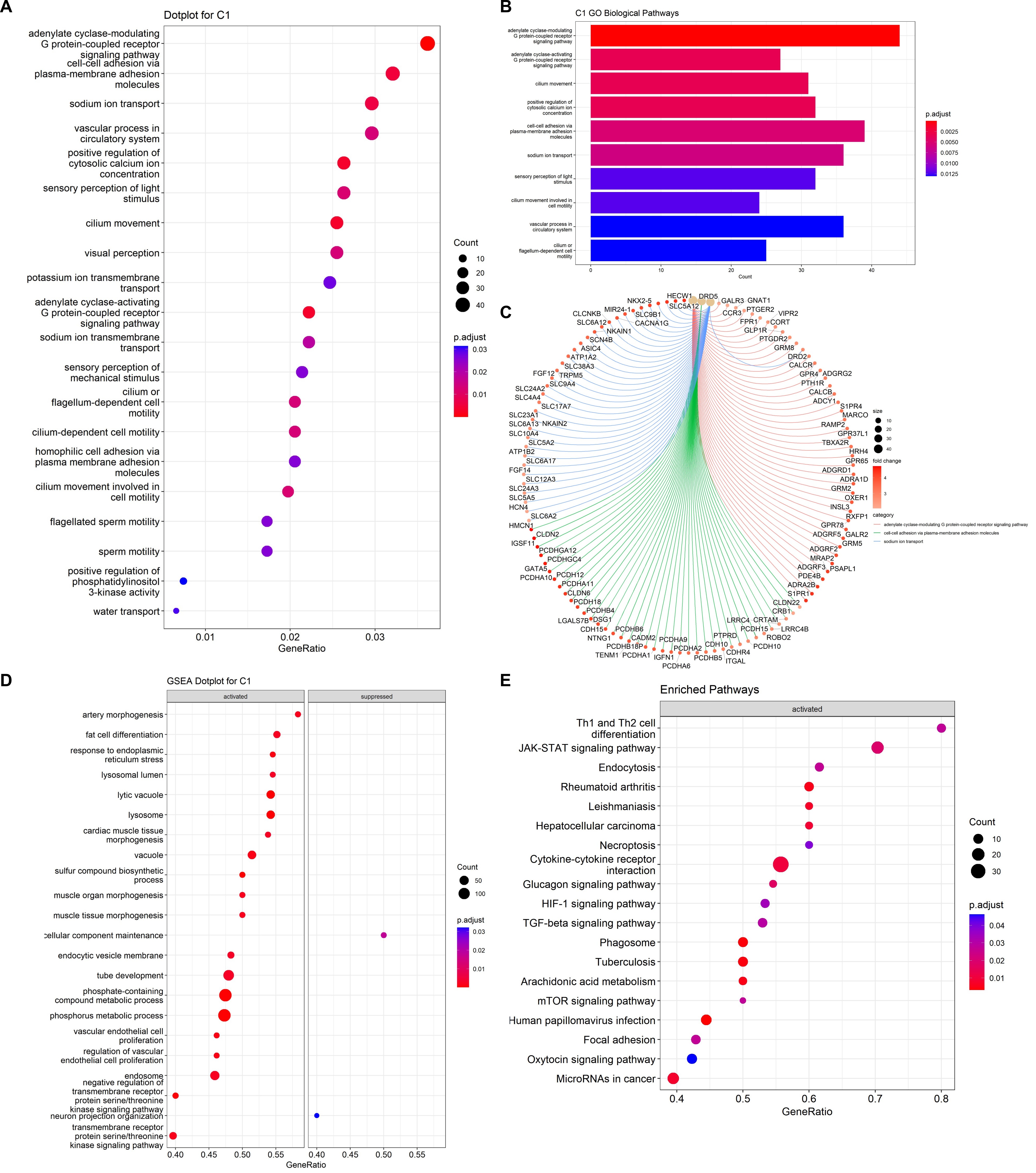

Supplement: Supplementary file 4 — Additional file 4. Figure S4. Differential gene expression analysis of C1.GO biological pathways overrepresented in C1 showed adenylate cyclase-modulating G protein-coupled receptor signaling pathway, cell–cell adhesion via plasma-membrane adhesion molecules and sodium ion transport.An enrichment map visualizing the connections between enriched GO terms in the C1 cluster.GSEA dot plot displayed activated and suppressed pathways for C1.Enriched pathways in C1 identified through KEGG pathway analysis [file 12935_2025_3671_MOESM4_ESM.jpg]

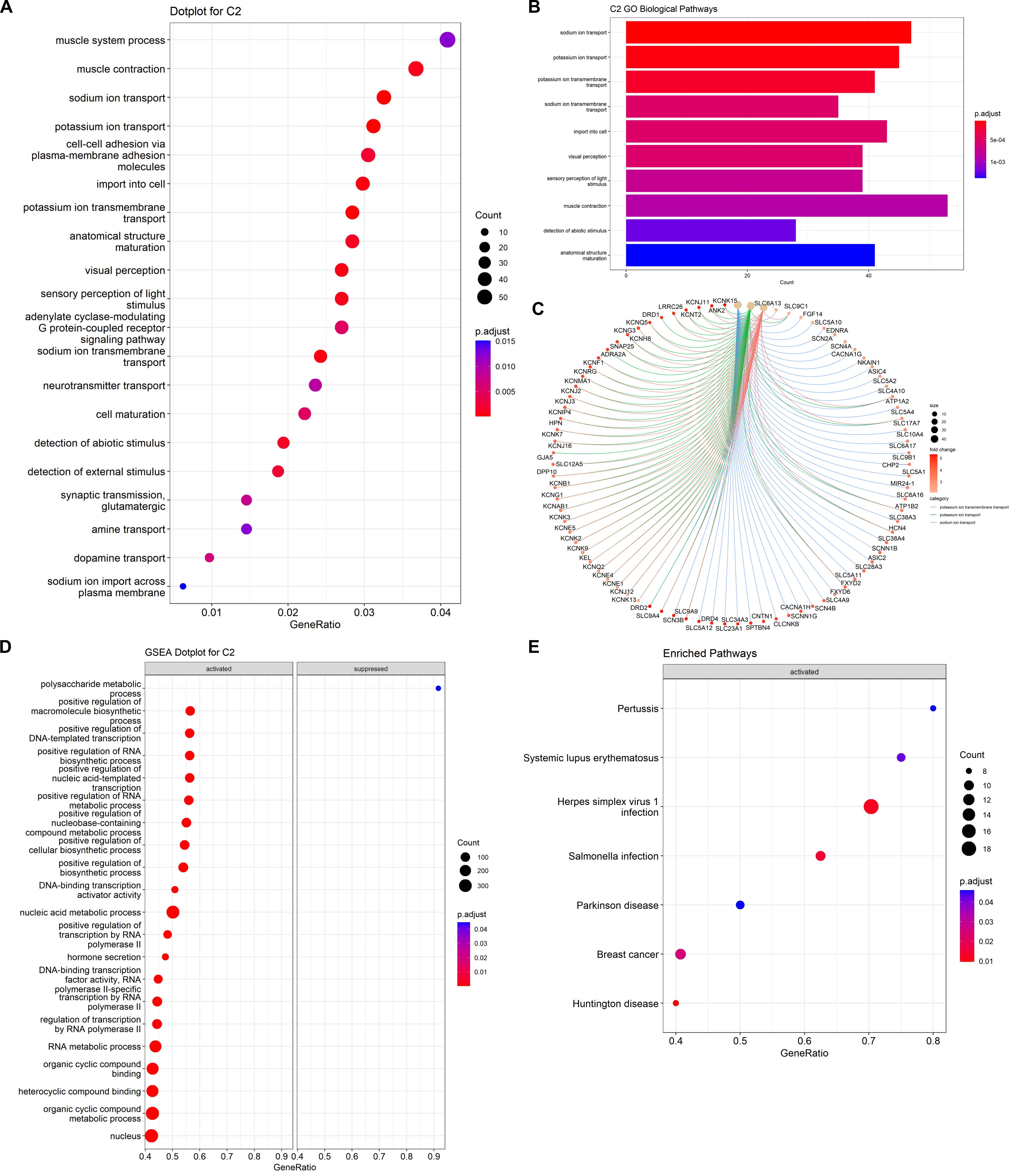

Supplement: Supplementary file 5 — Additional file 5. Figure S5. Differential gene expression analysis of C2.GO biological pathways overrepresented in C2 showed muscle contraction, sodium ion transport and potassium ion transport.An enrichment map visualizing the connections between enriched GO terms in the C2 cluster.GSEA dot plot displayed activated and suppressed pathways for C2.Enriched pathways in C2 using KEGG pathways [file 12935_2025_3671_MOESM5_ESM.jpg]

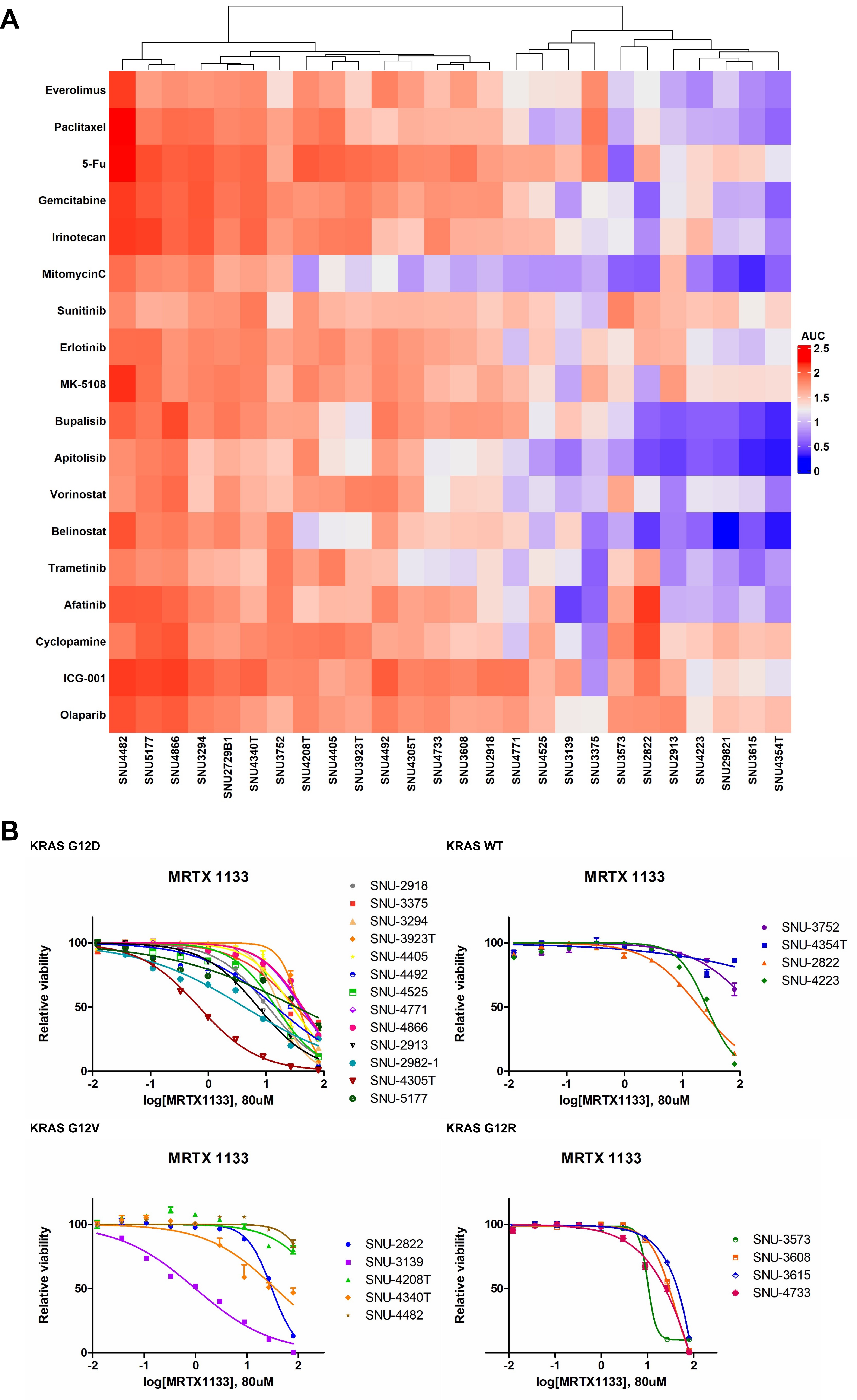

Supplement: Supplementary file 6 — Additional file 6. Figure S6. Drug sensitivity profiling and MRTX1133 response in PCCLs.AUC values for 18 anti-cancer drugs tested across PCCLs. Higher sensitivity is represented in blue, while lower sensitivity is represented in red.Viability curves of cell lines treated with MRTX1133 stratified by KRAS mutations status. Relative cell viability is plotted against the logarithm of drug concentration [file 12935_2025_3671_MOESM6_ESM.jpg]

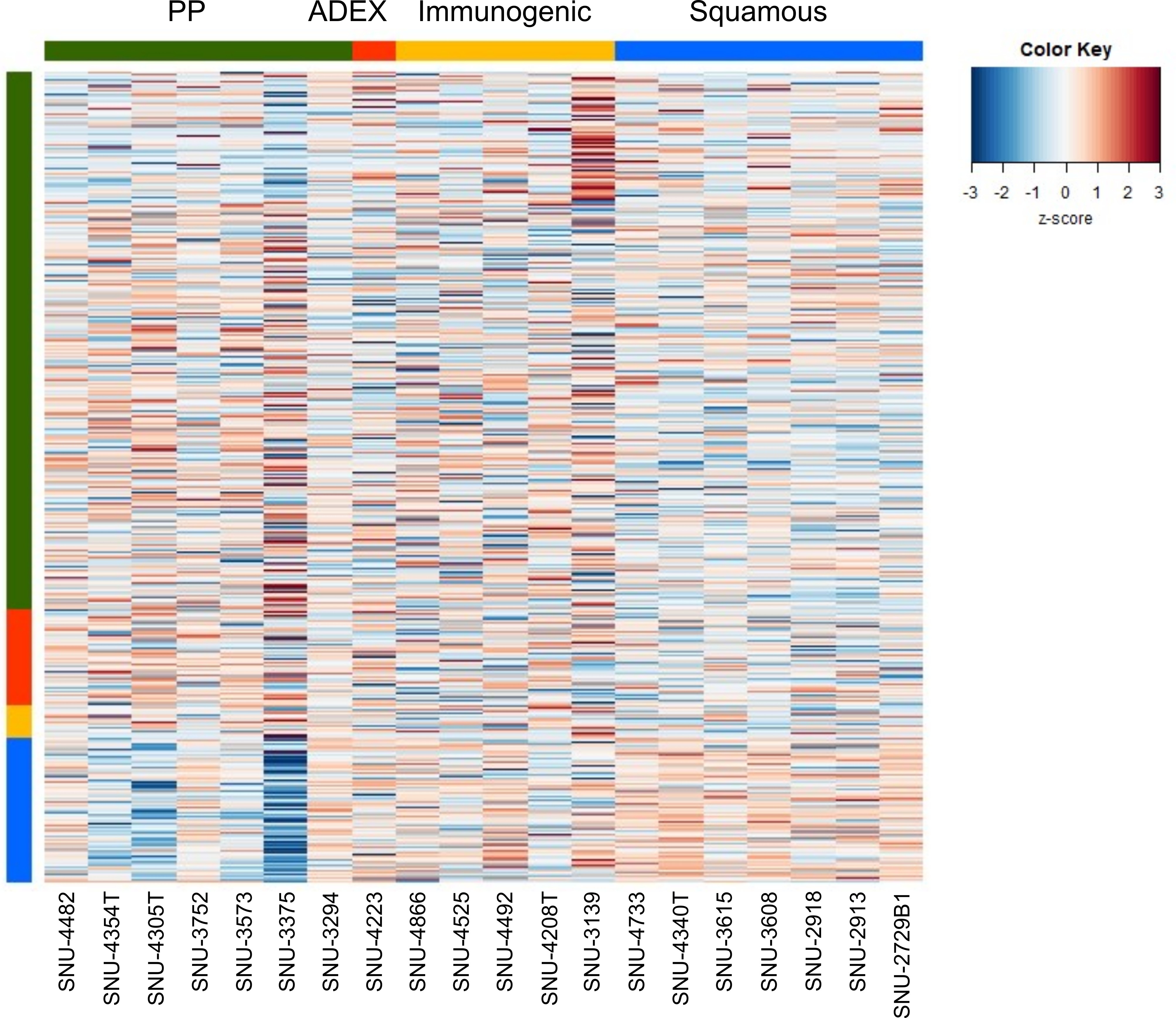

Supplement: Supplementary file 7 — Additional file 7. Figure S7. Heatmap of gene expression in PCCLs categorized by Bailey subtypes [file 12935_2025_3671_MOESM7_ESM.jpg]

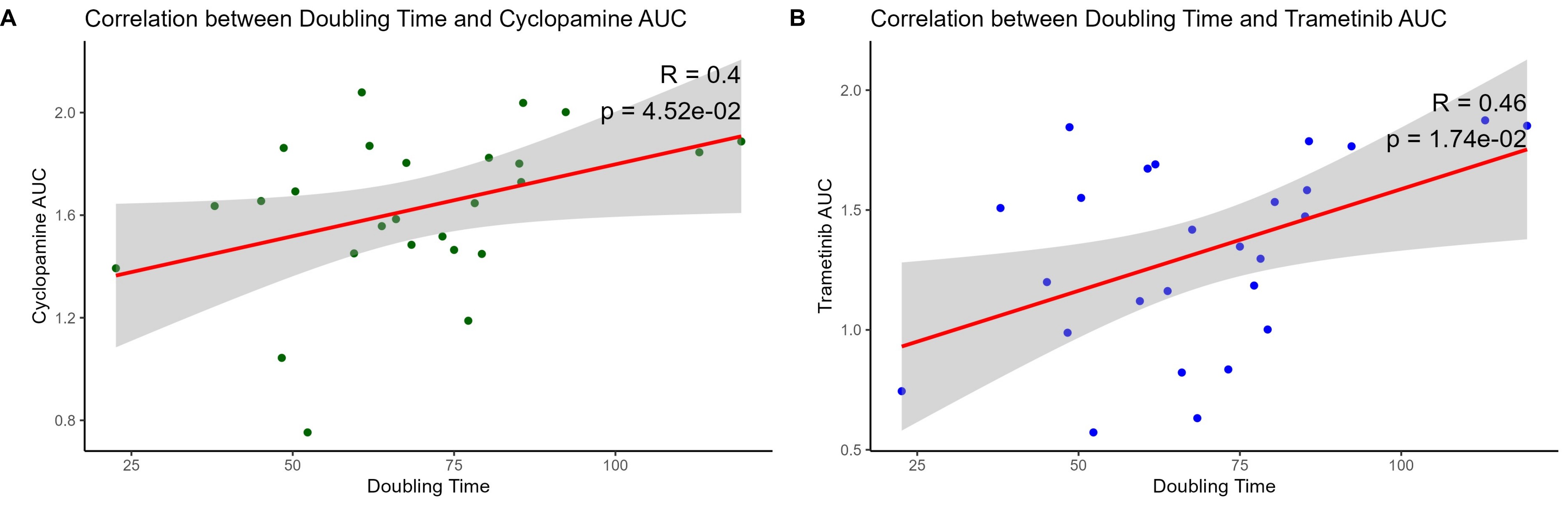

Supplement: Supplementary file 8 — Additional file 8. Figure S8.Correlation between doubling time and Cyclopamine sensitivity, represented by the AUC. A positive correlation was observed.Correlation between doubling time and Trametinib sensitivity, represented by the AUC [file 12935_2025_3671_MOESM8_ESM.jpg]
